# Circularly polarized light detection using chiral hybrid perovskite

**DOI:** 10.1038/s41467-019-09942-z

**Published:** 2019-04-26

**Authors:** Chao Chen, Liang Gao, Wanru Gao, Cong Ge, Xinyuan Du, Zha Li, Ying Yang, Guangda Niu, Jiang Tang

**Affiliations:** 1 Sargent joint research center, Wuhan National Laboratory for Optoelectronics, Huazhong University of Science and Technology, Wuhan, 430074 Hubei P. R. China; 2 School of Optical and Electronic Information, Huazhong University of Science and Technology, Wuhan, 430074 Hubei P. R. China

**Keywords:** Electronic devices, Optical materials and structures

## Abstract

Circularly polarized light (CPL) detection is required in various fields such as drug screening, security surveillance and quantum optics. Conventionally, CPL photodetector needs the installation of optical elements, imposing difficulties for integrated and flexible devices. The established CPL detectors without optical elements rely on chiral organic semiconductor and metal metamaterials, but they suffer from extremely low responsivity. Organic-inorganic hybrid materials combine CPL-sensitive absorption induced by chiral organics and efficient charge transport of inorganic frameworks, providing an option for direct CPL detection. Here we report the CPL detector using chiral organic-inorganic hybrid perovskites, and obtain a device with responsivity of 797 mA W^-1^, detectivity of 7.1 × 10^11^ Jones, 3-dB frequency of 150 Hz and one-month stability, a competitive combined feature for circularly polarized light detection. Thanks to the solution processing, we further demonstrate flexible devices on polyethylene terephthalate substrate with comparable performance.

## Abstract (web summary)

Optics-free circularly-polarized light detection has suffered from extremely low responsivity. Here Chen et al. demonstrate chiral organic–inorganic hybrid perovskite based detectors to distinguish circularly-polarized light with high responsivity of 797 mA/W.

## Introduction

Polarization, the same as intensity and wavelength, is a fundamental property of light. Circularly polarized light (CPL) is a light that the electric field has the constant magnitude but its direction continuously rotates in a plane with a steady rate. When interacting with a substance, the change of polarization state reflects the properties of this substance such as chemical composition, structure symmetry, surface roughness and so on^1^. Thus, a sensitive photodetector not only reveals the morphology of the target, but also provides valuable information regarding its chemical and physical nature. In fact, many invertebrates such as mantis shrimp, cuttlefish, bees and crickets have developed a CPL-sensitive system in their retina^2^ to enhance visual contrast and differentiate substances for better navigation, communication and other applications. Polarization sensitive photodetector is thus highly valuable for application in many fields such as drug screening^3^, security surveillance^4^, remote sensing^5^ and quantum optics^6^. Because of the lack of intrinsic chirality in inorganic semiconductors such as Si and InGaAs, conventional CPL-sensitive photodetector inevitably requires the installation of a quarter-wave plate and a linear polarizer on top of the non-chiral photodetectors. This strategy enables sensitive discrimination of polarized light despite at the price of increased cost and imposing some difficulties for integration and miniaturization.

One obvious solution is to find chiral sensitive materials that could naturally differentiate left-handed circularly polarized (LCP) and right-handed circularly polarized (RCP) light. We name the detection of CPL by the photodetector itself as direct CPL detection, while those require the assistance of optical elements as indirect CPL detection. Direct CPL detection has intrinsic advantages over indirect strategies for large-scale integration and for flexible electronics because of the removal of bulky wave plates (normally made of birefringent quartz, mica crystals and polymer sheet). Yet, to our best knowledge, there are only two papers in the literature reporting direct CPL detection to date^7,8^. In 2013, A. Campbell et al. demonstrated a field-effect phototransistor employing chiral helicene for direct CPL detection with responsivity of 10 mA W^−1^ at a wavelength of 365 nm^7^. Two years later, J. Valentine et al. successfully built silver metamaterials with chiral structure and obtained distinctive optical absorption toward RCP and LCP light at a communication interesting wavelength of 1340 nm^8^. They further showed direct CPL detection based on hot electron injection but suffered from low responsivity of 2.2 mA W^−1^ mainly limited by the ultrafast decay of hot electrons in surface plasmons. Since responsibility reflects how sensitive a photodetector responds toward incident light and the commercial Si photodiode has a responsivity of approximately 1 A W^−1^ (ref. ^9^), such a low responsivity of the available direct CPL detectors will certainly impede their practical applications, especially for weak CPL light detection and high-definition imaging.

We pursued therefore the goal of CPL detection via seeking other suitable chiral materials. For a direct CPL photodetector to perform well, the absorber needs to combine handedness sensitive optical absorption and efficient charge transport simultaneously. Organic molecules are preferred for optical absorption because chiral organic compounds with strong circular dichroism (CD) are abundant in nature. For example, all amino acids show chirality except glycine^10^; the large biological molecules, such as proteins, DNA and RNA, and many natural products, such as chlorophyll copper sodium^11^ and carvone^12^, are known for their CPL-sensitive absorption. On the other hand, inorganic compounds generally enjoy high carrier mobility thanks to the transport within their bands. Combination of organic molecules with inorganic compounds could be an ideal strategy to overcome their own shortcomings and obtain a synergy for sensitive CPL detection. Decoration of inorganic quantum dots (QDs) with chiral organic ligands have been successfully engineered in the past decade to obtain CPL-sensitive optical absorption^13^, however, no such film has been applied for direct CPL detection possibly due to the insulating organic ligands that impede efficient charge transfer between QDs^14^. An alternative, probably more promising, is to construct organic–inorganic hybrid semiconductors. One prominent example is the recently emerged lead halide perovskites, which possess low-cost solution processing^15^, strong defect tolerance^16^, and long carrier diffusion length^17^, and have demonstrated outstanding performance for photovoltaics^15^, light emitting diodes^18^ and visible^19^, and X-ray^20,21^ detector application. Very recently, by simply replacing the methylammonium with chiral amines or even other chiral organic molecules into A sites^22–24^, as-synthesized chiral halide perovskites not only possess handedness sensitive absorption by the chiral organics^25^, but also inherit the exceptional material and optoelectronic properties of the inorganic (PbI_6_)^4−^ octahedral framework, a perfect combination for direct CPL detection.

**Fig. 1:**
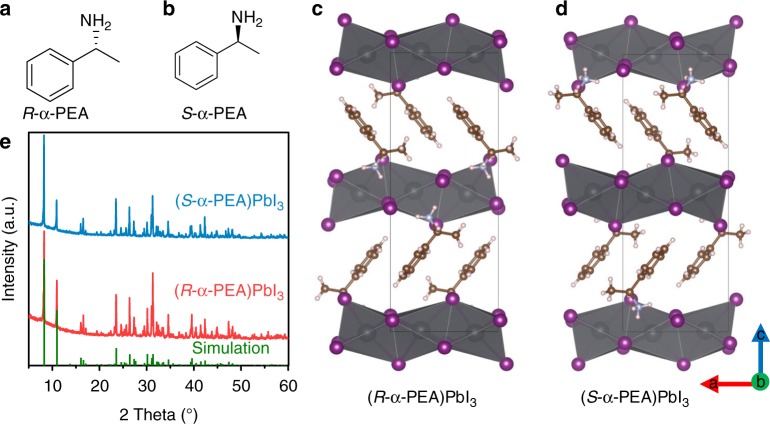
The crystal structures and properties of our chiral perovskites. **a**, **b** Molecular structures of *R*- and *S*-α-PEA. **c**, **d** The crystal structure of (*R*- and *S*-α-PEA)PbI_3_. The gray, rose red, golden yellow, light blue, and fresh pink dots stand for Pb, I, C, N, and H atoms, respectively. **e** Powder XRD patterns of (*R*- and *S*-α-PEA)PbI_3_. The simulated XRD peaks are shown as a comparison

We showcase here the synthesis of chiral organic–inorganic hybrid perovskites and their successful application for direct CPL photodetectors. We choose chiral α-phenylethylamine (α-PEA) to synthesize chiral perovskites, because the π bond in the benzene ring of α-PEA would facilitate columbic interaction between chiral amines and (PbI_6_)^4−^ matrix, enhancing CPL-sensitive absorption, and also because right-hand (*R*-α-PEA, Fig. 1a) and left-hand (*S*-α-PEA, Fig. 1b) enantiomers are commercially available with low cost^26^. One additional advantage is that α-phenylethylamine hydroiodide [(α-PEA)I] has good solubility in dimethylformamide (DMF) and dimethylsulfoxide (DMSO) and hence permits easy single crystal growth and thin film fabrication.

## Result

### Single crystal fabrication

Single crystals of ABX_3_-type perovskite (*R*- and *S*-α-PEA)PbI_3_ were first produced by the inverse temperature crystallization method^27^. Three millimoles (α-PEA)I and 3 mmol PbI_2_ were dissolved in 45% HI solution (6 mL) at 90 °C. Then the solution was slowly cooled from 90 to 40 °C with a cooling rate of 5 °C per hour. After 10 h, some yellow crystals formed from the solution (Supplementary Fig. 1), which were collected for further analysis. Single crystal diffraction experiments were performed; crystal structures were solved by Olex2 software^28^ (Supplementary Table 1, Fig. 1c, d) and are consistent with literature^25^. The crystals exhibit one-dimensional (1D) structure with face sharing (PbI_6_)^4−^ octahedral chains surrounded by the chiral (α-PEA)^+^ cations. The (*R*- and *S*-α-PEA)PbI_3_ have a chiral space group of P2_1_2_1_2_1_ assigned to orthorhombic crystal system^25^, so they have only two-fold screw symmetry. Note that the chiral α-PEA stays closely to the vertex of (PbI_6_)^4−^ octahedron enabling strong interaction between chiral organics and achiral inorganic parts. We failed to synthesize the racemic single crystal [denoted as (*rac*-α-PEA)PbI_3_)] because in our growing conditions only (*rac*-α-PEA)_2_PbI_4_ was produced. To confirm the structures, we also carried out powder X-ray diffraction (XRD) analysis on the ground powder of (*R*- and *S*-PEA)PbI_3_ single crystals, and all diffraction peaks were well consistent with the simulated results (Fig. 1e). An expanded XRD pattern is shown in Supplementary Fig. 2 for better peak resolution. The strongest diffraction peak at 8.27° is assigned to (002) crystal face where (PbI_6_)^4−^ octahedral chains stack in parallel.

### Device design and film fabrication

For a photoconductive CPL photodetector, maximized value of Δ*n*/*n* (where Δ*n* and *n* are equal to *n*_L_ − *n*_R_ and (*n*_L_ + *n*_R_)/2, respectively; *n*_L_ and *n*_R_ are carrier concentration generated by LCP and RCP light illumination, respectively) should be pursued to enhance device sensitivity. Δ*n*/*n* as a function of (α-PEA)PbI_3_ thickness (*d*) can be addressed as (Supplementary Note 1):

1 $$\frac{{\Delta n}}{n} \approx \frac{{\Delta \alpha d}}{{{\mathrm{exp}}\left( {\alpha _{\mathrm{L}}d} \right) - 1}}$$

where *α*_L_ and *α*_R_ are the absorption coefficient of LCP and RCP light, respectively; Δ*α* is equal to *α*_L_ − *α*_R_. When the active layer is too thick, the exponential term of exp(*α*_L_*d*) in the denominator can dramatically reduce the value of Δ*n*/*n* (Supplementary Fig. 3). This means all incident LCP and RCP photons can be fully absorbed, resulting in no photocurrent difference between LCP and RCP illumination. Therefore, the thick single crystal (approximately 1 mm) is unsuitable for CPL photodetector. Herein, films with appropriate thickness (equal to 1/*α*_L_ as shown in Supplementary Fig. 3) are expected to obtain maximum Δ*n*/*n*.

**Fig. 2:**
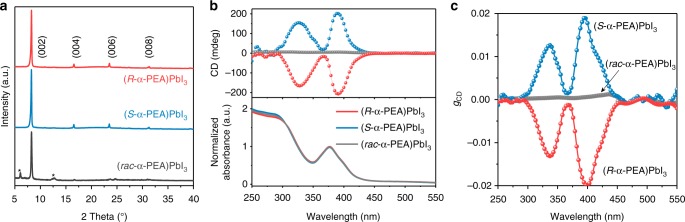
The characterization of (α-PEA)PbI_3_ films. **a** XRD patterns of (*R*-, *S*-, and *rac*-α-PEA)PbI_3_ films. The peaks marked with stars can be assigned to the (002) and (004) peaks of (α-PEA)_2_PbI_4_ phase. **b** CD and absorbance spectra of highly-oriented (*R*-, *S*-, and *rac*-α-PEA)PbI_3_ films. **c** The *g*_CD_ spectra of (*R*-, *S*-, and *rac*-α-PEA)PbI_3_ films

Luckily, the low-dimensional perovskites are prone to assemble themselves into highly-oriented and high-quality films by spin-coating technique^29^. The precursor was prepared by dissolving (*R-* or *S*-α-PEA)PbI_3_ single crystal in DMF solution with the concentration of 0.3 mol L^−1^ to fabricate (*R-* or *S*-α-PEA)PbI_3_ films. The racemic precursor (0.3 mol L^−1^) prepared by mixing (*R*- and *S*-α-PEA)PbI_3_ precursor with a ratio of 1:1 was used for (*rac*-α-PEA)PbI_3_ films. XRD patterns of (*R*-, *S*- and *rac*-α-PEA)PbI_3_ films showed sharp diffraction peaks which can be assigned to (002), (004), (006), and (008) planes (Fig. 2a), indicating the highly preferred orientation along *c*-axis, the axis perpendicular to the plane of the film (Supplementary Fig. 4). The small full width at half maximum of these peaks also revealed high crystallinity. Two impurity peaks traceable to (002) and (004) of (α-PEA)_2_PbI_4_ phase^22^ appeared in the (*rac*-α-PEA)PbI_3_ films due to the competitive formation of this secondary phase. Atomic force microscope measurement suggested that (*rac*-α-PEA)PbI_3_ film was likely composed of mixed domains of (*R*-α-PEA)PbI_3_ and (*S*-α-PEA)PbI_3_ (Supplementary Fig. 5). All the films showed strong excitonic absorption at 374 nm (Fig. 2b), a signature of the strong quantum confinement experienced by the (PbI_6_)^4−^ chains in these materials with 1D crystal structure^30^. Both (*R-* or *S*-α-PEA)PbI_3_ films showed strong CD signals while (*rac*-α-PEA)PbI_3_ film showed featureless absorption in the CD spectra. For the chiral perovskites, the CD spectra showed two intense peaks at 328 and 392 nm with the corresponding intensity of 183 and 210 mdeg, respectively. Ben-Moshe et al. discovered that the CD spectra can be calculated on the basis of the split excitonic transition states^31,32^, and each CD signal should have a corresponding excitonic transition^22^, implying two excitonic transitions in (α-PEA)PbI_3_. The multiple excitonic transitions were also observed in other low-dimensional perovskites (α-PEA)_2_PbI_4_^22^ and (C_4_H_9_NH_3_)_2_PbBr_4_^33^. (*rac*-α-PEA)PbI_3_ exhibited no CD signal because of the racemic mixture nature. The anisotropy factor of CD (*g*_CD_, calculated as CD/(32980 × absorbance))^22^ at 392 nm is ~0.02 (Fig. 2c), which is three times higher than that of (α-PEA)_2_PbI_4_ perovskite^22^ and one order of magnitude larger than that of ordinary chiral QDs^34^. High-resolution polarized optical microscopy study (Supplementary Fig. 6) confirms that CD is mainly originated from chiral perovskites, however, the weak dependence of CD spectra on the rotation angle (Supplementary Fig. 7) suggests that linear birefringence or linear dichroism may also take effect to some extent.

### The origin of chirality in hybrid perovskites

Now we discuss the origin of chirality in our hybrid perovskites. First, the CD signal positions of (*R*- or *S*-α-PEA)PbI_3_ (328 nm and 392 nm) are significantly different from the CD position of α-PEA (259 nm, Supplementary Fig. 8). We thus propose that the chirality of the (*R*- and *S*-α-PEA)PbI_3_ perovskites comes from the chiral crystal structure (P2_1_2_1_2_1_ space group)^35^. From the view of quantum mechanics, the CD intensity is proportional to the rotational strength (**R**) as^32,36^:

2 $${\mathrm{CD}} \propto {\mathbf{R}} = {\mathrm{Im}}\left[ {\left\langle {{\mathrm{\Psi }}_{\mathrm{s}}{\mathrm{|}}{\hat{\mathbf{\mu }}}{\mathrm{|\Psi }}_{\mathrm{j}}} \right\rangle \cdot \left\langle {{\mathrm{\Psi }}_{\mathrm{j}}{\mathrm{|}}{\hat{\mathbf{m}}}{\mathrm{|\Psi }}_{\mathrm{s}}} \right\rangle } \right] = {\mathrm{Im}}\left[ {{\mathbf{\mu }}_{{\mathrm{sj}}} \cdot {\mathbf{m}}_{{\mathrm{js}}}} \right]$$

where Ψ_s_ and Ψ_j_ are the wave functions of the ground state and excited state; **μ**_sj_ and **m**_js_ are the electric and magnetic transition dipole moments, respectively. To ensure a nonzero CD, the **μ**_sj_ and **m**_js_ should be able to transform as the same irreducible representation (Supplementary Table 2)^23^. Only non-centrosymmetric chiral point groups satisfy the condition, therefore possessing **μ**_sj_ ≠ 0 and **m**_js_ ≠ 0 and exhibiting CD^23^. For achiral point groups, because of their either inversion symmetry or central symmetry element, **R** is equal to zero, hence there is no optical activity. As mentioned above, (*R*- or *S*-α-PEA)PbI_3_ is assigned to non-centrosymmetric chiral space group of P2_1_2_1_2_1_, corresponding to point group of **D**_2_. Therefore the (*R*- or *S*-α-PEA)PbI_3_ exhibit chirality.

### Device fabrication and performance

**Fig. 3:**
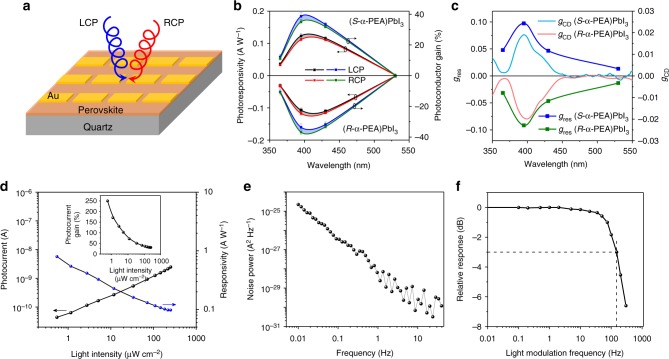
The device performance of (*R*- and *S*-α-PEA)PbI_3_ film photodetector. **a** Schematic diagram of our photodetector. **b** The responsivity and photoconductor gain of (*R*- and *S*-α-PEA)PbI_3_ device under LCP and RCP light at the wavelengths of 365, 395, 430, and 530 nm. The gray and cambridge blue area denotes the difference of responsivities and photoconductor gain under LCP and RCP irradiation, respectively, with identical intensity. **c** The wavelength dependent *g*_res_ spectra. The *g*_CD_ spectra are also shown here in order to facilitate comparison. **d** Light intensity dependent LDR and responsivity at 20 V under 395 nm irradiance. The inset is the light intensity dependent photoconductor gain. **e** Measured current noise at various frequencies. **f** Normalized response of the photodetector versus the input signal frequency. The -3-dB point is specified with the dash lines

We assembled the highly-oriented (α-PEA)PbI_3_ films into photodetector devices (Fig. 3a). Au electrodes defining a channel length of 10 μm were prepared by thermal evaporation. Before measurement, we carefully calibrated our system to assure the same intensity of LCP and RCP illumination during all measurements (Supplementary Fig. 9). Figure 3b showed the responsivity and photoconductor gain of (*R*- and *S*-α-PEA)PbI_3_ photodetectors under the CPL with the wavelengths of 365 nm (6.2 μW cm^−2^), 395 nm (5.0 μW cm^−2^), 430 nm (7.4 μW cm^−2^), and 530 nm (79 μW cm^−2^). The peak responsivity of our device can reach 0.12 A W^−1^ under 395 nm irradiation (corresponding to a photoconductor gain of 39.0%). Our devices demonstrated notably different responsivity between LCP and RCP at the wavelengths of 395 nm, however smaller difference at 365 nm and 430 nm, and nearly no difference at 530 nm, which are consistent with the CD and *g*_CD_ spectra. In analogy to the *g*_CD_, we defined the anisotropy factor of responsivity (*g*_res_, which describes the distinguishability to CPL) as:

3 $$g_{{\mathrm{res}}} = \frac{{2\left( {R_{\mathrm{L}} - R_{\mathrm{R}}} \right)}}{{R_{\mathrm{L}} + R_{\mathrm{R}}}}$$

where *R*_L_ and *R*_R_ are the responsivity under LCP and RCP illumination, respectively. As long as the conversion and collection efficiency of absorbed photons to electron-hole pairs is the same, *g*_res_ is in principle equal to *g*_CD_ (Supplementary Note 1). In our case, the maximum *g*_res_ is 0.1 at the wavelength of 395 nm, higher than *g*_CD_ of 0.02 at 395 nm. This is possibly caused by the spin-dependent carrier transport and collection^37^. Patrick Odenthal et al. has reported that the CPL can generate spin-electron in CH_3_NH_3_PbI_3_ perovskite due to the spin-dependent optical selection rules^38^. In addition, caused by the large Rashba splitting in perovskite and ruled by the angular momentum conservation^23,39^, the spin-up and spin-down electrons (holes) stay at different conduction band minimum (valence band maximum)^23,38^. This may affect the spin-dependent carrier transport via carrier effective mass. The anisotropy factor of responsivity in our device is inferior to the chiral photodetector employing plasmonic metamaterials, which enjoys an extremely large *g*_CD_ of 0.9 and *g*_res_ of 1.09 (the 1.09 value is calculated by ourselves based on the reported *R*_L_/*R*_R_ of 3.4)^7,8^. Obviously, the *g*_CD_ of our device should be further improved in the future.

We also tested the linear dynamic region (LDR) of our device under unpolarized 395 nm light with light intensity ranging from 0.05 to 252 μW cm^−2^. We did not choose 395 nm CPL light for LDR measurement because the maximum intensity of our light source is limited to 5.0 μW cm^−2^ due to optical attenuation by the waveplate. The device exhibited wide LDR within nearly three orders of magnitude (Fig. 3d). The light intensity dependent responsivity and photoconductor gain were illustrated in Fig. 3d. The responsivity can reach 797 mA W^−1^ (corresponding to a photoconductor gain of 253%), which is nearly two order of magnitude larger than the chiral molecule based^7^ and metamaterial based^8^ CPL photodetectors. The frequency dependent noise spectrum in dark is showed in Fig. 3e, which showed strong 1/*f* characteristics because its polycrystalline nature causes substantial carrier scattering at the grain boundaries^40^. Combining the measured noise current (*i*_n_) at 1 Hz (2.5 × 10^−29^ A^2^ Hz^−1^) and the corresponding responsivity (797 mA W^−1^), we can calculate the detectivity (D*) through $D^ {\ast} = R\sqrt A /i_n$ (where *R* is responsivity, *A* is the effective area of the detector)^41^ as 7.1 × 10^11^ Jones. The detectivity is impressive as commercial Si photodiode has a polarization-insensitive detectivity of approximately 10^12^ Jones^41^. The 3-dB frequency, defined as the frequency at which response dropped to half of the initial value, was approximately 150 Hz for 395 nm response, faster than the 30 frame per second required for imaging application. All combined, our direct CPL photodetector shows high sensitivity, low noise and fast response, a balanced and competitive performance for polarization sensitive imaging, manifesting the advantage of using chiral perovskites for direct CPL detection. Compared to the performance of CH_3_NH_3_PbX_3_-based CPL-insensitive photodetectors (e.g. best responsibility of approximately 10 AW^-1^, *D*^∗^ of 10^12^–10^14^ Jones)^19,42^, there is a large room for further optimization of our CPL-sensitive photodetectors, which could be achieved by identifying better chiral perovskites, improving film quality and exploring appropriate device architecture.

**Fig. 4:**
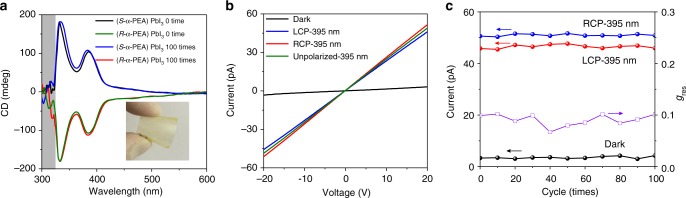
Device performance of (α-PEA)PbI_3_ films on flexible PET substrate. **a** The CD spectra of (*R*- and *S*-α-PEA)PbI_3_ films on PET substrates without bending and with 100-times bending. Inset is the digital photograph of the film on PET substrate. **b** The current-voltage curves of (*R*-α-PEA)PbI_3_ device under dark, LCP-395, RCP-395, and unpolarized-395 nm light illumination. The light intensity was 5 μW cm^−2^. **c** The current under dark, LCP-395 and RCP-395 nm irradiation as well as *g*_res_ as a function of folding cycles

The direct CPL based on chiral materials holds promise for flexible optoelectronic devices. Enabled by the low-temperature solution process, here we fabricate our direct CPL photodetector onto flexible polyethylene terephthalate (PET) substrate. Optical measurement of our perovskite film revealed two CD peaks at 330 and 385 nm (Fig. 4a), consistent with previous results on quartz. The noisy CD spectra from 300 to 320 nm were caused by the parasitic absorption of PET substrate. Encouragingly, the device also demonstrated CPL detection capability (Fig. 4b) with comparable *g*_res_ of 0.08 (0.1 for the device on quartz substrate). The responsivity under LCP-395 nm, RCP-395 nm and unpolarized-395 nm light illumination (5.0 μW cm^−2^) are 0.090, 0.098, and 0.094 A W^−1^ (0.12 A W^−1^ for the device on quartz substrate under 5.0 μW cm^−2^). The current under dark, LCP-395 nm and RCP-395 nm light illumination as well as the *g*_res_ as a function of folding times are shown in Fig. 4c. Both the CD spectra, dark current and photocurrent, and the anisotropy factor of responsivity (*g*_res_) exhibited negligible degradation upon 100 times of bending, confirming the robustness of our chiral perovskite films. We envision our work would enable many unexplored applications for flexible and/or wearable electronics.

**Fig. 5:**
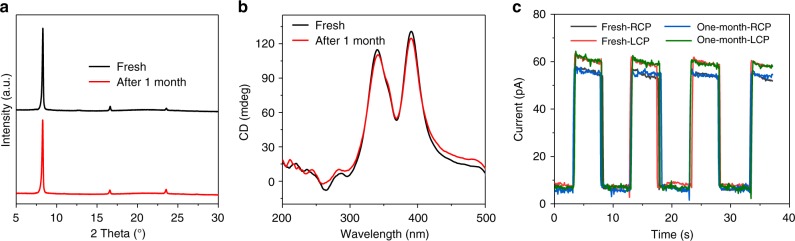
The ambient stability of (*R*-α-PEA)PbI_3_ films. **a** XRD patterns, **b** CD spectra, and **c** Photoresponse of fresh and 1-month aged (*S*-α-PEA)PbI_3_ films under LCP and RCP 395 nm light. (*S*-α-PEA)PbI_3_ films are stored in a laboratory environment without any encapsulation

We further monitored the XRD pattern, CD spectra and electrical stability of our (*S*-α-PEA)PbI_3_ film after stored in ambient without any encapsulation for 1 month to check its stability (Fig. 5). All of these characterizations revealed negligible degradation after the ambient storage. The device also exhibited no degradation when continuously operated for 2 h (Supplementary Fig. 10). Our chiral perovskites demonstrated similar stability compared to low-dimensional achiral perovskites^22,30^. Recent calculations revealed that the energy required to remove a phenethylammonium iodide from low-dimensional perovskite is 0.36 eV higher than that of methylammonium iodide from 3D perovskite, reducing the desorption rate of organic molecules by 6 orders of magnitude^30^. This explains the environmental stability of our chiral hybrid perovskites.

## Discussion

The moderate *g*_res_ of our CPL detector need improvement for practical application. According to Eqs. (1) and (3), improvement in *g*_CD_ represents a straightforward strategy to boost *g*_res_. We thus propose the following potential approaches to strengthen the chiral sensitivity of our hybrid perovskites CPL detector: (i) Seek organic amines with multiple chiral sites. Chirality transfer accounts for the chirality of perovskites^23^; amine with multiple chiral sites (such as threonine, isoleucine, 1,2-diphenylethylenediamine, etc.) could be explored into halide perovskites. (ii) Engineer a chiral octahedron and inorganic framework without mirror symmetry or centro-symmetry. Small B-site cation (such as Cu^2+^, Ge^2+^, Sb^3+^, etc.) and mixed anions at X-site (Cl^−^, Br^−^, I^−^, SCN^−^, etc.) can significantly distort the (BX_6_)^4−^ octahedron and potentially produce strong chirality and improve *g*_CD_ in principle. (iii) Produce mesoscopically chiral ordering. Similar to metamaterials^8^, structured halide perovskite films with characteristic lengths comparable to the wavelength of light enjoy strong chiroptical effect, leading to the improvement of *g*_res_.

In summary, we successfully fabricated a direct CPL detector using chiral organic–inorganic hybrid (α-PEA)PbI_3_ perovskites with an impressive responsivity of 795 mA W^−1^. This device showed a measured detectivity of 7.1 × 10^11^ Jones, 3-dB frequency of 150 Hz and high stability, a balanced and competitive device performance for potential polarization sensitive imaging applications. We also demonstrated the flexible CPL detector on PET substrate with high performance and robust flexibility. Moreover, chiral organic–inorganic hybrids combine the polarization sensitive absorption of chiral organics and efficient charge transfer of inorganics, providing a rich family of candidate materials for direct CPL detection. Such hybrids include halide perovskites incorporating other chiral amines such as benzedrine, (2-naphthyl)ethanammonium and sec-butylamine into A sites, and the large family of metal-organic frameworks (MOFs) materials containing chiral organics at the framework. We envision that direct CPL detection without optical elements would open an exciting realm for the easy realization of miniaturized polarization sensitive imaging systems.

## Methods

### Materials

*R*-α-phenylethylamine (*R*-α-PEA, 99.0% gas chromatography purity, 98% enantiomeric excess), *S*-α- phenylethylamine (*S*-α-PEA, 99.0% gas chromatography purity), N,N-anhydrous dimethylformamide (DMF, 99.8%), lead iodide (PbI_2_, 99.9%) were purchased from Aladdin (Shanghai, China). Forty-five percent aqueous hydriodic acid (HI) solution (99.95%) was purchased from Sinopharm Chemical Reagent Co., Ltd. (China).

### Synthesis of (*R*- and *S*-α-PEA)I

(*R*-α-PEA)I (or (*S*-α-PEA)I) was prepared by slowly mixing *R*-α-PEA (or *S*-α-PEA) (5.1 mL) with HI (5.8 mL) in a 1:1 molar ratio under continuous stirring at 0 °C for 2 h. (*R*-α-PEA)I (or (*S*-α-PEA)I) was then crystallized by removing the solvent from a rotary evaporator at 80 °C for 4 h, washed three times in diethyl ether and collected by filtering. The white crystal was dried in vacuum for 24 h and then kept in a dark and dry environment for further use.

### Growth of (*R*- and *S*-α-PEA)PbI_3_ single crystals

0.75 g (3 mmol) of (*R*-α-PEA)I (or (*S*-α-PEA)I) and 0.69 g (3 mmol) of PbI_2_ were fully dissolved in 6 ml of a 45% HI solution at 90 °C. Then 1 mL simethicone was added to protect the precursor from air. The solution was slowly cooled from 90 to 40 °C with a cooling rate of 5 °C per hour. Many yellow crystals produced after 10 h. These crystals were collected and washed by hexane to remove simethicone from the surface.

### Fabrication of (α-PEA)PbI3 film

Quartz glass and polyethylene terephthalate (PET) were used as the substrate for rigid and flexible devices. Both substrates with the desired dimensions were cleaned in an ultrasonic cleaner using detergent, acetone, 2-propanol, ethanol, and deionized water in sequence for 30 min of each. Next, the substrate surface was cleaned by an ultraviolet-ozone cleaner for 10 min. The precursor solutions for the chiral perovskites were prepared by dissolving the (*R*- or *S*-α-PEA)PbI_3_ single-crystal powders in DMF with the desired concentration (0.3 mol L^–1^). To form the films, 80 μL of the precursor solution was spread on the cleaned surface of the substrate, and then spun at 1000 rpm for 10 s, 3000 rpm for 70 s. Finally, the as-fabricated film was annealed at 95 °C for 10 min on a hot-plate to induce crystallization.

### Device preparation

Planar structured photoconductive detectors were fabricated by thermally evaporating Au electrodes onto (*R*- or *S*-α-PEA)PbI_3_ films, and the channel between neighboring electrodes had a width of 0.5 mm and a length of 10 μm. The thermal evaporation was conducted using the electron beam and resistance evaporation thin film deposition system (Beijing Technol Science Co. Ltd.) under a vacuum pressure 5 × 10^−3^ Pa.

### Material characterization

Powder XRD measurements were performed by grinding (*R*- and *S*-α-PEA)PbI_3_ into fine powders in a mortar using a Philips diffractometer (X pert pro MRD) with a step of 0.017^o^ and step time of 15.05 s from 5° to 40°. The lines are Cu K_α1_ and Cu K_α2_ with wavelengths of 1.540598 Å and 1.544426 Å, respectively. The CD and extinction spectra were collected using a CD spectrometer (J-815, JASCO, Easton, MD, USA) at room temperature. The background was air, and the spectra were obtained at a scan rate of 200 nm min^−1^, with the data pitch being 1 nm. For single crystal diffraction measurement, diffraction data were collected on a Bruker SMART 1K CCD with standard PILATUS3 R 200K detector. The X-ray source was generated by Cu MicroMax-003 microfocus sealed tube. The collection method is *ω*-scans of 0.5° width. Crystal structure was first analyzed by direct method with SHELXS solution program and then refined by SHELXL using least-squares method. All of nonhydrogen atoms’ positions were located using difference Fourier method. Crystal data are listed in Supplementary Table 1. Powder diffraction XRD pattern of (α-PEA)PbI_3_ was simulated by software VESTA. For *µτ* test, the dark and light (395 nm, 37 μW cm^−2^) current at 1 V to 100 V were measured by Agilent B1500A.

### Detector performance measurement

The current-voltage and current-time curves were measured using Agilent B1500A in an electromagnetically shielded probe station. The light was generated from LEDs with wavelength of 365 nm (Thorlabs M365L2), 395 nm (Thorlabs M395L2), 430 nm (Thorlabs M430L3) and 530 nm (Thorlabs M450L3). Circularly polarized light was obtained by a linear polarizer (Thorlabs, LPVIS200-100-A) and quarter-wave plate (Thorlabs, AQWP05M-600). The intensity of LCP and RCP light was calibrated by standard Si detector (Newport 818-UV/DB) before each measurement with extreme caution (Supplementary Fig. 9). The 3-dB and noise power were measured by a pre-amplifier (SR570) and a lock-in amplifier (SR850, Stanford Research Systems).

## Supplementary information

Supplementary Information

## Data Availability

The data that support the findings of this study are available from the corresponding author on request.
