# Supplementary material for: Circularly polarized light detection using chiral hybrid perovskite
Source: Nat Commun. 2019 Apr 26;10:1927. doi: 10.1038/s41467-019-09942-z (PMC6486588; doi:10.1038/s41467-019-09942-z)
Supplement: Supplementary file 1 — Supplementary Information [file 41467_2019_9942_MOESM1_ESM.pdf]

Supplementary Information

Sensitive circularly polarized-light detection using chiral hybrid perovskite

Chen et al.

Supplementary Figures

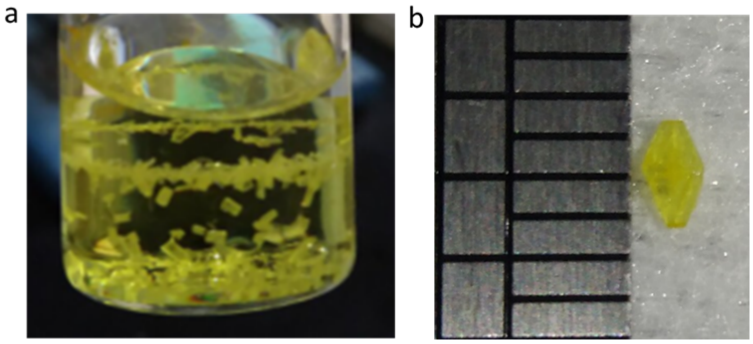

Supplementary Figure 1 | The photograph of (*R*-α-PEA)PbI_3 single crystal. a, The formed yellow crystals on the well of bottole. **b,** One of the single crystal in the bottle with the size of 3 mm×2 mm×1 mm.

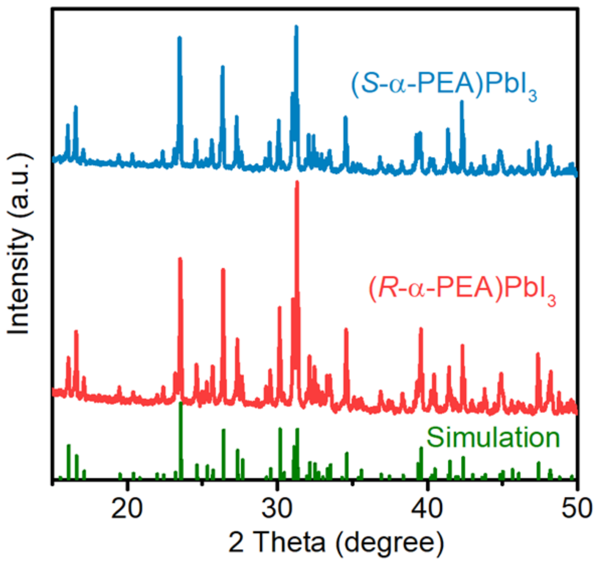

Supplementary Figure 2 | The expanded version of XRD patterns from 15 to 50 degree. All the experimental XRD peaks are consistent with the simulated results.

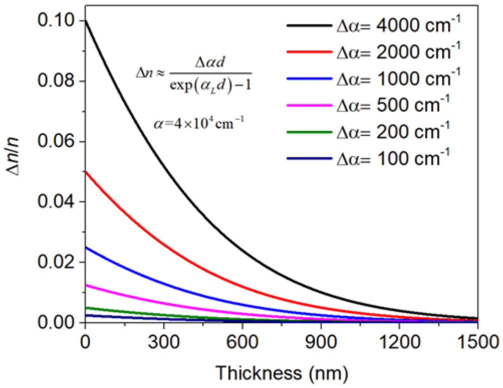

Supplementary Figure 3 | **$\Delta n/n$ as a function of film thickness.** The $\Delta n/n$ exponentially decays with the increase of film thickness.

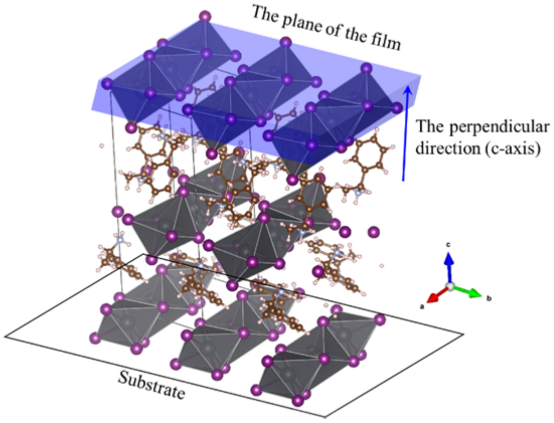

Supplementary Figure 4 | The c-axis direction of (R-α-PEA)PbI_3 thin films. The crystallographic *c*-axis direction is perpendicular to the plane of the (002)-oriented film.

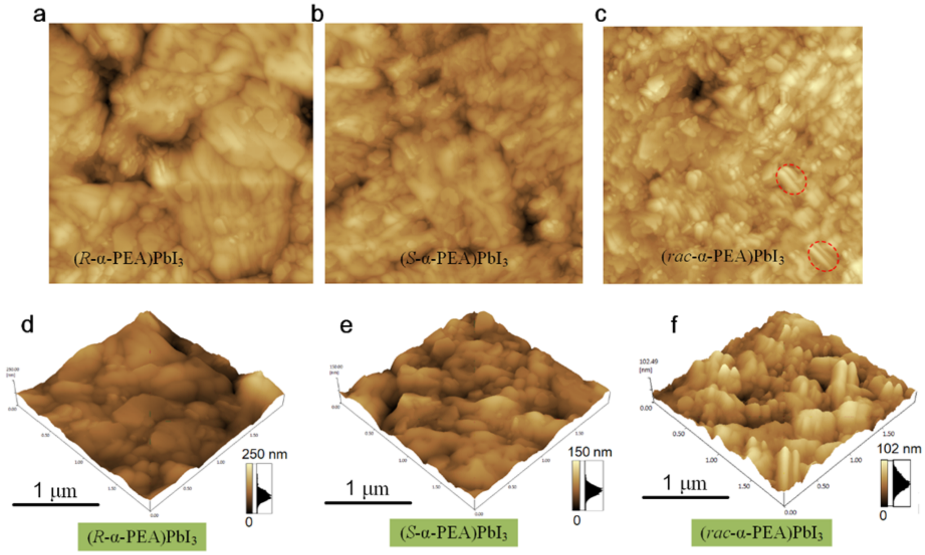

Supplementary Figure 5 | Surface morphology of (*R*-, *S* and *rac*-α-PEA)PbI_3 thin films. The thin-film morphologies of (α-PEA)PbI_3 were studied by atomic force microscope (AFM). **a,b,d,e,** The pure chiral (*R*- and *S*-α-PEA)PbI_3 films showed uniform grains. **c,f,** For (*rac*-α-PEA)PbI_3 films, one grain is often accompanied with another one (red dash circles in panel **c**). We hypothesize that the two grains correspond to perovskite domains of opposite chirality, which explains in part the observed achiral nature of the racemic film.

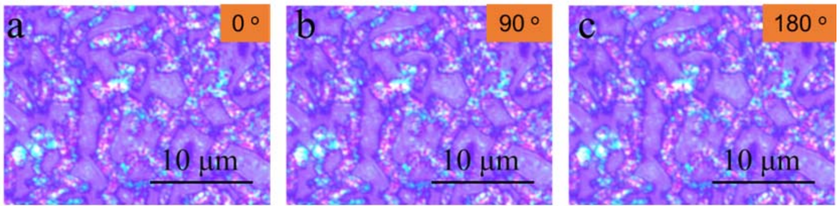

Supplementary Figure 6 | The images of (*R*-α-PEA)PbI_3 films under high-resolution polarized optical microscopy. The images of (*R*-α-PEA)PbI_3 films were performed on a high-resolution polarized optical microscopy (LEICA DM4000M). No distinct difference is observed on the morphology of the highly-oriented films with the rotation of polarizer, confirming the CD signal probably do not come from linear dichroism.

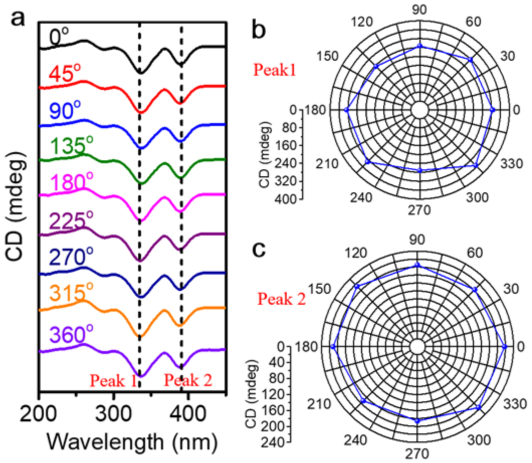

Supplementary Figure 7 | The CD spectra of (*R*-α-PEA)PbI_3 films with different rotation angles. a, The CD spectra of (*R*-α-PEA)PbI_3 films with different rotation angles. **b,c,** The intensity of CD signal as a function of rotation angles at Peak 1 and Peak 2. Such observation confirms that CD signal is mainly originated from chiral perovskites, yet the weak dependence of CD signal intensity on the rotation angle suggests that the linear birefringence or linear dichroism may also at play.

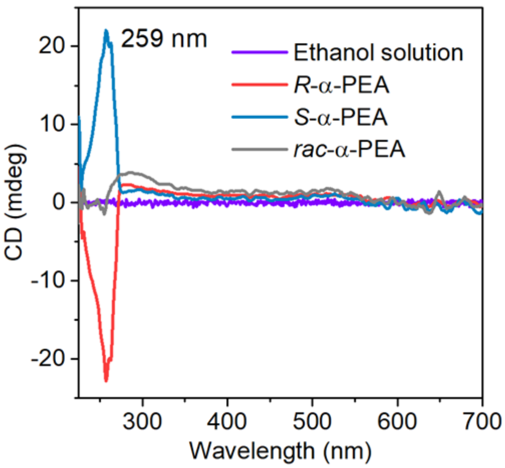

Supplementary Figure 8 | The CD spectra of *R*-, S- and *rac*-α-PEA. *R*-, S- and *rac*-α-PEA were diluted in ethanol solution with volume ratio of 1:100. The CD peaks of *R*- and *S*-α-PEA were at 259 nm.

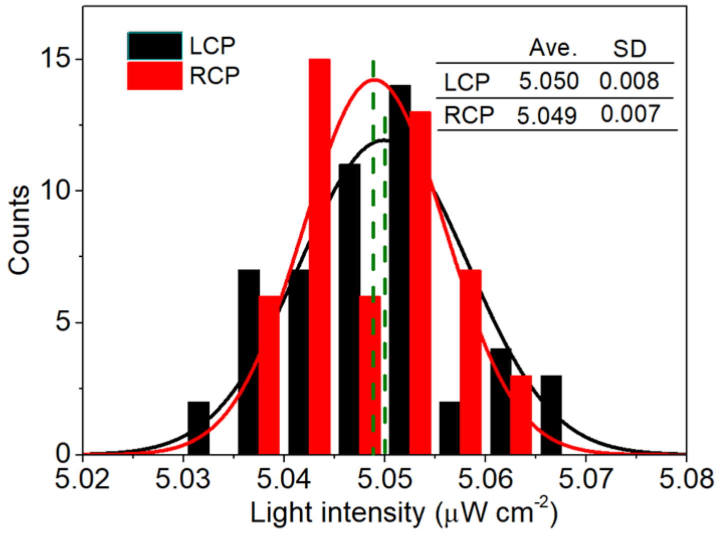

Supplementary Figure 9 | The statistical graph of LCP and RCP light intensity. The LCP and RCP light intensity are 5.050±0.008 μW cm^{-2} and 5.049±0.007 μW cm^{-2}, respectively. 100 data (50 for LCP; 50 for RCP) are counted. Firstly, we waited 15 min to ensure the intensity of light source (395 nm LED) was stable. Then we put the linear polarizer and quarter-wave plate between the light source and a standard Si detector (Newport 818-UV/DB). Next, we switched the CPL from LCP to RCP (then from RCP to LCP…) for 50 cycles, and monitored the light intensity before each switch. At last, we obtained 50 data for LCP and the other 50 data for RCP. The entire testing process was performed in an electromagnetic shielding box under dark condition (except the 395 nm light source). As shown in the statistical graph (Fig. S8), the light intensity of LCP and RCP are 5.050±0.008 μW cm^{-2} and 5.049±0.007 μW cm^{-2}, respectively. The relative standard deviation (defined as the ratio of the standard deviation to the mean) of LCP and RCP light intensity are 0.16% and 0.14%, which are two orders of magnitude smaller than the g_{res} (~0.1). In conclusion, the different responsibilities to LCP and RCP light are caused by the chiral device, rather than the light intensity error

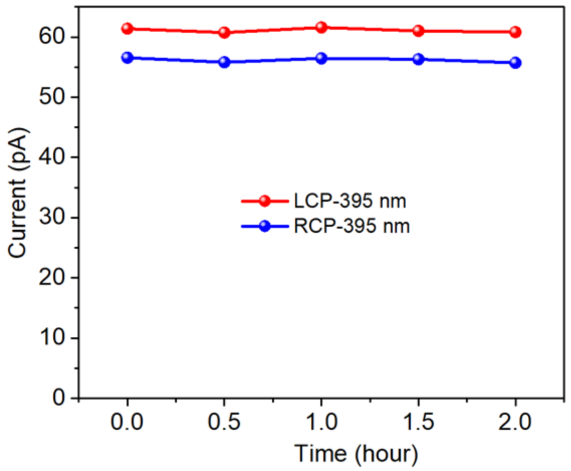

Supplementary Figure 10 | The stability of device under operational condition. The operational stability of our (*S*-α-PEA)PbI_3 photodetector was measured under continuous 395 nm light illumination. The device also exhibited no degradation for 2 hours. The test was performed on device without any encapsulation in an electromagnetic shielding box at room temperature. The device was continuously exposed in 395 nm light for 2 hours. We measured the device performance under LCP and RCP illumination per 0.5 hour.

Supplementary Tables

Supplementary Table 1 | The crystal data of (*R*- and *S*-α-PEA)PbI_3. Crystal structure was analyzed by direct method with SHELXS solution program [1]. The grogram of SHELXL with least-squares methods was used to refine the structure. All of nonhydrogen atom's positions were located using difference Fourier method. The obtained crystal structures are consistent with the literature [2].

Empirical Formula	R-($C_8H_{12}N$)PbI_3	S-($C_8H_{12}N$)PbI_3
M	710.08	710.08
Crystal system	Orthorhombic	Orthorhombic
Space group (NO.)	$P2_12_12_1$ (19)	$P2_12_12_1$ (19)
T/K	296	296
a/Å	8.0856(1)	8.0862(1)
b/Å	8.7046(1)	8.7027(1)
c/Å	21.3556(2)	21.3567(3)
α /°	90	90
β /°	90	90
γ /°	90	90
V/Å^3	1503.05(3)	1502.92(3)
Z	4	4
d_{calc}/g cm^{-3}	3.138	3.138
Radiation wavelength	Cu kα (λ =1.54184)	Cu kα (λ =1.54184)
μ/mm^{-1}	69.938	69.951
F(000)	1232	1221
Scan range (θ)/°	4.14–73.71	4.14–73.47
Index ranges	$-9 \leq h \leq 9$, $-10 \leq k \leq 9$, $-26 \leq l \leq 24$	$-5 \leq h \leq 9$, $-10 \leq k \leq 10$, $-26 \leq l \leq 22$
Total reflections	5508	4575
Unique reflections R(int)	6542 [R_{int}=0.0904, R_{sigma}= 0.0508]	5180 [R_{int}=0.0922, R_{sigma}= 0.0474]
Parameters	120	56
Flack parameter	0.05(3)	-0.01(7)

Supplementary Table 2 | The relationship between point group, electric dipole moment ($\boldsymbol{\mu}_{sj}$), magnetic dipole moment ($\mathbf{m}_{js}$), rotational strength (***R***) and circular dichroism (CD) [3].

Point group	$\boldsymbol{\mu}_{sj}$	$\mathbf{m}_{js}$	Angle between $\boldsymbol{\mu}_{sj}$ and $\mathbf{m}_{js}$	***R*** (CD)	Chiral perovskite
$\boldsymbol{C}_{\mathbf{i}}$, $\boldsymbol{C}_{\mathbf{nh}}$, $\boldsymbol{D}_{\mathbf{nh}}$, $\boldsymbol{D}_{\mathbf{nd}}$ (n≠2), $\boldsymbol{S}_{\mathbf{2n}}$ (n odd), $\boldsymbol{O}_{\mathbf{h}}$, $\boldsymbol{T}_{\mathbf{d}}$, $\boldsymbol{I}_{\mathbf{h}}$	$\boldsymbol{\mu}_{sj}\neq0$	$\mathbf{m}_{js}=0$	/	**R**=0 (CD=0)	/
***C*s**, $\boldsymbol{C}_{\mathbf{nv}}$, $\boldsymbol{D}_{\mathbf{2d}}$, $\boldsymbol{S}_{\mathbf{2n}}$ (n even)	$\boldsymbol{\mu}_{sj}\neq0$	$\mathbf{m}_{js}\neq0$	90 °	**R**=0 (CD=0)	/
$\boldsymbol{C}_{\mathbf{n}}$, $\boldsymbol{D}_{\mathbf{n}}$, ***O***, ***T***, ***I***	$\boldsymbol{\mu}_{sj}\neq0$	$\mathbf{m}_{js}\neq0$	0° or 180°	**R**≠0 (CD≠0)	(*R*-α-PEA)PbI_3 (*S*-α-PEA)PbI_3: $\mathbf{D_2}$

Supplementary Note

Supplementary Note 1 | The calculation model for g_{res} and g_{CD}.

Both the photon number of LCP and RCP are n_0. The absorbed number by chiral film is n_L and n_R.

$$\begin{cases} n_L = n_0\left(1-e^{-\alpha_L d}\right) \\ n_R = n_0\left(1-e^{-\alpha_R d}\right) \end{cases} \tag{1}$$

Where α_L and α_R are the absorption coefficient of LCP and RCP, d is the thickness of active layer. The value of $\Delta n/n$ (where Δn and n are equal to n_L-n_R and $(n_L+n_R)/2$, respectively) as a function of thickness (d) can be addressed as:

$$\begin{aligned} \frac{\Delta n}{n} &= \frac{2(n_L - n_R)}{(n_L + n_R)} \\ &= \frac{2n_0\left(1-\exp\left(-\alpha_L d\right)\right) - 2n_0\left(1-\exp\left(-\alpha_R d\right)\right)}{n_0\left(1-\exp\left(-\alpha_L d\right)\right) + n_0\left(1-\exp\left(-\alpha_R d\right)\right)} \\ &= \frac{2\exp\left(-\alpha_R d\right) - 2\exp\left(-\alpha_L d\right)}{2-\exp\left(-\alpha_L d\right) - \exp\left(-\alpha_R d\right)} \\ &= \frac{2\exp\left(\Delta\alpha d\right) - 2}{2\exp\left(\alpha_L d\right) - 1 - \exp\left(\Delta\alpha d\right)} \end{aligned} \tag{2}$$

where $\Delta\alpha$ is equal to α_L-α_R. Because $\Delta\alpha d$ is far less than 1, $\exp(\Delta\alpha d)$ is approximately equal to $1+\Delta\alpha d$. Then we can obtain that

$$\frac{\Delta n}{n} \approx \frac{\Delta\alpha d}{\exp\left(\alpha_L d\right) - 1} \tag{3}$$

Fixing α_L as 4×10^4 cm^{-1} and changing $\Delta\alpha$ from 100 to 4000 cm^{-1}, we can plot the $\Delta n/n$ against thickness of the film as Supplementary Fig. 3.

If the conversion and collection efficiency of absorbed photons to electron-hole pairs are the same for both (R-α-PEA)PbI_3 and (S-α-PEA)PbI_3, the g_{res} is equal to $\Delta n/n$. When $\alpha_L d$ is not so large compared to 1, g_{res} is approximately equal to g_{CD}.

$$\begin{aligned} g_{res} &= \frac{\Delta n}{n} \approx \frac{\Delta\alpha d}{\exp\left(\alpha_L d\right) - 1} \\ &< \frac{\Delta\alpha d}{\alpha_L d} = \frac{\Delta\alpha}{\alpha_L} \approx g_{CD} \end{aligned} \tag{4}$$

Supplementary References

1 Dolomanov, O. V. *et al.* OLEX2: a complete structure solution, refinement and analysis program. *J. Appl. Crystallogr.* **42**, 339 (2009).

2 Billing, D. G. & Lemmerer, A. Synthesis and crystal structures of inorganic–organic hybrids incorporating an aromatic amine with a chiral functional group. *CrystEngComm* **8**, 686 (2006).

3 Long, G. *et al.* Spin control in reduced-dimensional chiral perovskites. *Nat. Photonics* **12**, 528 (2018).
